# The theoretical cognitive process of visualization for science education

**DOI:** 10.1186/2193-1801-3-184

**Published:** 2014-04-10

**Authors:** Lindelani E Mnguni

**Affiliations:** Centre for Health Sciences Education, School of Clinical Medicine, University of the Witwatersrand, Johannesburg, South Africa

**Keywords:** Conceptualization of visual models, Externalization of visual models, Internalization of visual models, Multimedia, Science education, Visualization

## Abstract

The use of visual models such as pictures, diagrams and animations in science education is increasing. This is because of the complex nature associated with the concepts in the field. Students, especially entrant students, often report misconceptions and learning difficulties associated with various concepts especially those that exist at a microscopic level, such as DNA, the gene and meiosis as well as those that exist in relatively large time scales such as evolution. However the role of visual literacy in the construction of knowledge in science education has not been investigated much. This article explores the theoretical process of visualization answering the question “*how can visual literacy be understood based on the theoretical cognitive process of visualization in order to inform the understanding, teaching and studying of visual literacy in science education*?” Based on various theories on cognitive processes during learning for science and general education the author argues that the theoretical process of visualization consists of three stages, namely, Internalization of Visual Models, Conceptualization of Visual Models and Externalization of Visual Models. The application of this theoretical cognitive process of visualization and the stages of visualization in science education are discussed.

## Introduction

The past few decades have seen an explosion in the integration of technology and e-learning in science education. As part of this, students are expected to develop a number of competencies in order to work effectively in this technology-driven education. These competencies include development of the science competences (Turner & Dankoski 2008), 21st century skills (Arsad et al. 2011), content literacy (McKenna and Robinson 1990), academic communication literacy (Spektor-Levy et al. 2008), science literacy (Van Eijck and Roth 2010) and visual literacy (Bottomley et al. 2006). Visual literacy is one of the most critical competencies particular for students who are taught content knowledge such as molecular medicine, pathology and molecular biology because concepts (e.g. DNA, RNA and proteins) and phenomena (e.g. metabolic pathways) exist at complex microscopic levels which cannot be visualized with a naked eye. Visual models such as diagrams and animations are then used to represent these phenomena at a larger scale so as to assist students with construction of content knowledge (Dori and Barak 2001). However, Schönborn and Anderson (2010) argue that students and teachers need to develop visualization skills in order to work effectively with visual models. There is however a dearth of research regarding the nature of visualizations skills and visual literacy for science education. To this end, Schönborn and Anderson (2009) present factors that affect students’ ability to interpret visual models in biochemistry. They further present cognitive skills that encompass visual literacy in biochemistry (Schönborn and Anderson 2010). However there remains a gap in literature regarding the nature of visual literacy based on the cognitive process of visualization (Mnguni 2007).

The aim of this article therefore is to explore the theoretical process of visualization based on various studies on general cognitive processes with specific reference to molecular biology and science education. This paper does not present primary empirical data but theorizes the process of visualization by blending what other researchers have presented on cognitive processes in various other contexts. The question being answered is “*how can visual literacy be understood based on the theoretical cognitive process of visualization in order to inform the understanding, teaching and studying of visual literacy in science education*?” To respond to this question, various theories related to cognitive processing of information as documented in literature will be explored in an attempt to present a model that could be used to study visual literacy empirically within science education. It is because of the intricate nature of cognitive processes that the author believes that it is necessary to first present a reasonable theoretical basis on which empirical studies can be based. Two critical aspects are discussed in the article, namely, construction of knowledge using visual models as well as the theoretical cognitive process of visualization.

### Construction of knowledge using visual models

**Figure 1:**
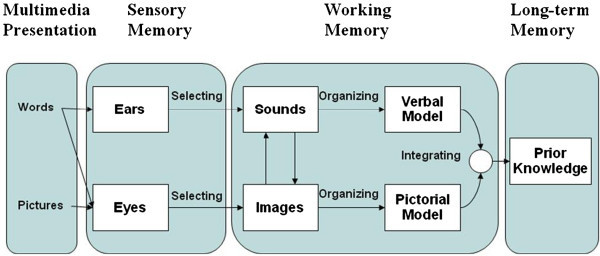
**An illustration of the cognitive theory of multimedia learning (adapted from Mayer** 2003 **).**

According to Mayer (2002) learning from visual models is a cognitive process that involves a number of mental processes as explained in the cognitive theory of multimedia learning. According to this theory during the learning process, external pictures first enter the cognitive system through the eyes (Figure 1). The student then attends to some aspects of the visual model which leads to the construction of a mental pictorial image within working memory. Following subsequent construction of mental images, the student arranges the set of images into a coherent mental representation called a pictorial model (Figure 1). The process involves the selection, organisation and integration of images and is commonly referred to as visuo*-*spatial thinking (Mayer 2003).

Mayer’s (2003) cognitive theory of multimedia learning is related to a constructivist epistemology of learning. According to constructivism, students actively develop their own understanding of the world, rather than having such understanding delivered to them (Thompson 1995; von Glasersfeld 1995). Such an outlook requires students to be active participants in the learning process, rather than merely absorbing the information presented to them in its entirety. Thompson (1995) suggests that students select and transform the information, construct hypotheses, and make decisions, based on an already existing cognitive structure. It is important to note that during cognitive processing of information, students tend to select information which is easiest to comprehend and manage mentally (Thompson 1995). Constructed information is then stored in the long term memory as mental schema. Students attempt to access these mental schema when stimulated to do so. This may include attempting to re-produce mental schema as external visual models (Brill et al. 2000). Communicating one’s thoughts through visual models can also include drawing on paper, generating visual models on a computer, manipulating visual models with software tool and manipulating a visual model externally.

Emanating from the constructivist epistemology of learning and Mayer’s (2003) cognitive theory of multimedia learning, during learning the visualization process therefore consists of at least three stages, namely, comprehension of visual information, processing of this information in cognitive structures, and externalization of information as visual models. The author also posits using constructivism and the cognitive theory of multimedia learning, that visualization can be defined as the ability to select and effectively use a set of cognitive skills for perceiving, processing and produce visual models. These deductions are explored further in this article as the bases of the proposed theoretical cognitive process of visualization.

### The theoretical cognitive process of visualization

**Figure 2:**
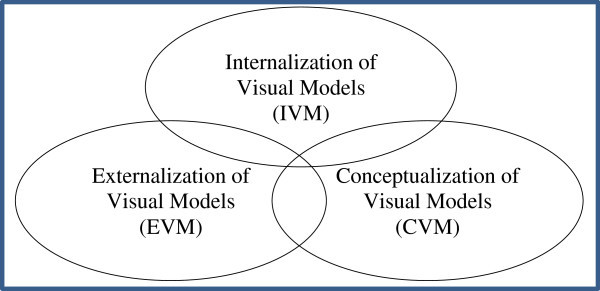
**The overlapping stages of the cognitive process of visualization.**

In relation to cognition, learning involves the internal (psychological) and external (physical) domains. Learning therefore involves processing of information as a way of interaction between these two domains. Cognitivism, constructivism and related theories such as the cognitive theory of multimedia learning address i) the input of information from the external world into the cognitive structures, ii) the cognitive processing of this information, and iii) the externalization of information from the mind to the world/environment. Based on this line of thinking therefore the author believes that the cognitive process of visualization can be divided into three non-linear overlapping stages; namely, Internalization of Visual Models (IVM), Conceptualization of Visual Models (CVM) and Externalization of Visual Models (EVM) (see Figure 2). In this model IVM refers to the process where sense organs, such as the eyes, work with the brain to “absorb” information from world (i.e. outside the body), whereas CVM is the process where meaning is made and during which cognitive visual models are constructed (Burton 2004). During CVM prior knowledge that is stored as cognitive visual models may be revised from the long term memory and reconstructed in the working memory based on new knowledge. EVM is the production of external visual models by way of expressing cognitive mental schema. Each of these stages is explored in detail in the following sections.

### Internalization of visual models (IVM)

The author believes that there are at least three levels of IVM, namely, low-level, middle level and high-level IVM (Healey 2005; van Schoren 2005). Low-level IVM involves mainly feature extraction whereas high level IVM involves a cognitively demanding process of concept formulation (Healey 2005).

Low-level IVM involves mainly pre-attentive visual tasks which require minimal cognitive effort to perform. These include target detection, region tracking and counting (Kawahara and Yokosawa 2001). For example, if a science student is asked to distinguish between an animal and a plant cell based on their organelles in a visual model, the student will only look for the presence or absence of structures such as the chloroplast and cell wall in the model. These tasks are performed by simply tracking the presence or absence of particular structures, such as when one detects the different texture boundaries between different items, the unique visual element on a background and/or when one estimates the number of items that contain a unique feature (Healey 2005).

Experimental evidence has also shown that performing pre-attentive tasks requires a relatively less degree of attention. In this regard, Stevenson and Roorda (2005) report that pre-attentive tasks can be performed in less than 200 to 250 milliseconds of viewing a visual model. These researchers argue that pre-attentive tasks can be performed in parallel with eye movements and with little or no effort to analyse them in the working memory. High-level IVM on the other hand occurs when a relatively high amount of cognitive effort is applied to internalizing visual information (Van Schoren 2005). This stage may be interconnected with the CVM stage as it may require an interpretation of visual models using prior knowledge (Healey 2005).

Once the information has been internalized, it is then transferred to the working memory for further processing in order to generate “meaning” by constructing mental schema. The accuracy of these mental schema will rely heavily on the precision with which the information is internalized. Koedinger and Anderson (1990) report that once information has been internalized it is organized into coherent patterns called chunks. This chunking may be followed by selecting and rearranging of information, cognitive processes that require a greater cognitive effort and attention.

**Figure 3:**
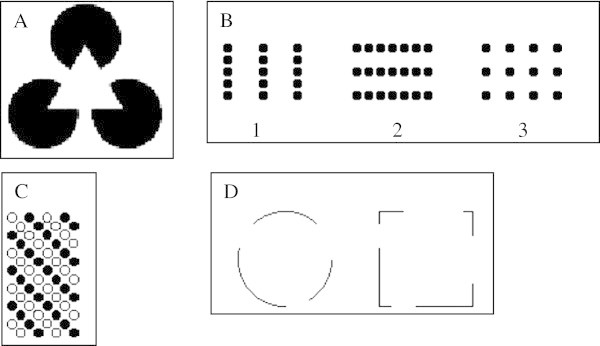
**The Gestalt principles.** In **A**, the principle of closure signifies our tendency to see complete figures even when part of the information is missing. In such a diagram we perceive three black circles covered by a white triangle, even though it could just as easily be three incomplete circles. In **B**, as stipulated by the principle of proximity, those parts that are closest together, we perceive the group (1) as three vertical lines of dots and the group (2) as three horizontal lines of dots. The dots in (3) are equally spaced and do not suggest an orientation. In **C**, the similarity principle suggests that we group together those parts that appear ‘similar’. Hence in **C**, we see separate white diagonal lines and black diagonal lines rather than vertical or horizontal lines of black and white dots. And in **D**, according to the principle of closure, we group together parts that give the appearance of closed shapes figures adapted from Mullet & Sano (1995).

Gestalt principles account for the manner in which visual models are processed cognitively during the high-level IVM, also called the post–attentive stage of IVM (Behrens 1984; Figure 2). Gestalt principles suggest that there are at least four main factors that determine how humans “chunk” information, namely, closure, proximity, similarity and simplicity (Figure 3). The *closure* principle suggests that the mind tends to complete figures even in cases where information is missing (see A in Figure 3). The principle of *proximity* (also referred to as the principle of *contiguity*) suggests that when visual features are placed closer to each other, they are perceived as a unit (see B in Figure 3; Mullet and Sano 1995). According to the *similarity* principle (see C in Figure 3), items that have commonalities such as shape, size, colour, texture and orientation are often grouped as belonging together (Mullet and Sano 1995). Finally, according to the *simplicity* principle, items are grouped together according to symmetry, regularity and smoothness (see C in Figure 3). All these principles reflect the behaviour of the cognitive system towards new visual information that has been perceived. Research in molecular biology has also confirmed the applicability of these Gestalt principles among students studying theoretical concepts. For example, Novick and Catley (2007) indicate that students have more learning difficulties with understanding phylogenetic ladders compared with phylogenetic trees. This is because the Gestalt principle of good continuation (proximity) obscures the hierarchical structure of ladders which is not the case with phylogenetic trees.

**Figure 4:**
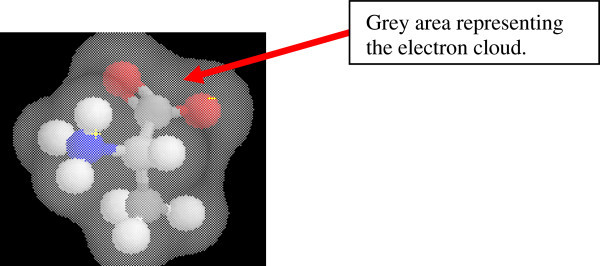
**ER in which students perceived the electron cloud as a background (adapted from Mnguni** 2007 **).**

Mnguni (2007) posits that a typical learning skill associated with IVM is “ability to comprehend the scientific meaning of the part of a visual model that lies behind objects that are in the foreground” such as the cytoplasm in a cell depicting organelles (Allen 1990). In his study Mnguni found that all participating students (100% of 106) did not see this background information in a visual model as meaningful or even associated with the rest of the image. An example of this was observed in a case where students did not comprehend the electron cloud as being part of the information presented in a model (Figure 4). Asked what is (and the significance of) the “grey area” in the model (Figure 4), one student (2P17) said, “*I don’t know, I am guessing…it’s a way of showing the amino acid…the background*”*.* This student seemed to lack conceptual meaning associated with the visual model presented. As a result, extracting meaningful knowledge (i.e. IVM) is limited and misconceptions develop.

On the contrary, another student recognized the actual background (highlighted in black in Figure 4) as part of the cell. This student (2P31) suggested that the black background (Figure 4) represented an “*empty space in the cell*”. This misconception may have arisen because the student felt a “need” to explain her thinking “scientifically” using relevant conceptual knowledge of the cell structure. By implication therefore, IVM is linked to the availability of conceptual knowledge from long term memory. Furthermore, Mnguni’s example demonstrates that IVM can be inter-linked with CVM as it requires prior conceptual knowledge.

### Conceptualization of visual models

Once information has been successfully internalized, CVM follows. According to Mast et al. (2003) CVM is where students rely on short and long term memories to conceptualize visual information by way of interpreting incoming visual information against prior knowledge as explained by constructivist theory (Mayer 2003; Thompson 1995).

A number of studies have been conducted to understand what exactly occurs when students view visual models leading to CVM. One such study was conducted by DeSantis and Housen (2000) who investigated how students process information when viewing artistic work. These scholars derived five stages of cognitive processing of visual models (CVM) (Table 1; Housen 1992). As presented in Table 1, the five stages are the “Accountive”, “Constructive”, “Classifying”, “Interpretive” and “Re-creative” stages (Housen 1992).

**Table 1:** **The Housen model used to characterize people into different stages of cognitive processing based on their actions as they view visual models (DeSantis and Housen** 2000 **, p. 13)**

Stage	Actions	Definition
I	**Accountive**	Use senses, memories, emotions and personal associations, to make concrete observations about the work which get woven into a narrative
II	**Constructive**	Use logical and accessible tools: their own IVMs, knowledge, values of their social, moral and conventional world. If work does not look the way it is “supposed to”—if craft, skill, technique, hard work, utility, and function are not evident— then work is “weird,” lacking, and of no value.
III	**Classifying**	Analytical and critical. Identify work as to place, school, style, time and provenance. Decode the work using library of facts and figures that they are ready and eager to expand.
IV	**Interpretive**	Seek a personal encounter with a work. Let the meaning of the work slowly unfold; appreciate the subtleties of line and shape and colour. Critical skills are put in the service of feelings and intuitions; let underlying meanings of the work—what it symbolizes—emerge. Each encounter with a work of art presents a chance for new comparisons, insights, and experiences. Knowing that the work of art’s identity and value are subject to reinterpretation, these students see their own processes subject to chance and change.
V	**Re-creative**	Have established a long history of viewing and reflecting. A familiar painting is like an old friend who is known intimately, yet full of surprise. Combines personal contemplation with views that broadly encompass universal concerns.

Stage I is the least cognitively demanding whereas stage V is the most demanding.

In the accountive stage, students conceptualize visual models based on what is known and also what is liked as found in their long term memory (DeSantis and Housen 2000; Housen 1992). In the constructive stage, students employ logical and accessible tools of knowledge to make judgements about the visual model (DeSantis and Housen 2000; Housen 1992). In this instance, should the image not fit what it should be like according to the student’s long term memory, then such an image makes no sense to the student. In the classifying stage students attempt to classify what is perceived into categories that occur in their memory (DeSantis and Housen 2000; Housen 1992). In the interpretive stage students allow the meaning of the work to unfold rather than them imposing it (DeSantis and Housen 2000; Housen 1992). And finally, in the re-creative stage students allow an establishment of varying meanings each time they view an image, even if they had a previous meaning (DeSantis and Housen 2000; Housen 1992). In this regard prior knowledge is used to make new discoveries about the image at hand.

According to DeSantis and Housen (2000), students tend to move from one stage to the next based on factors such as gain of new knowledge and experience in the field. This gradual development in the way people conceptualize visual models is in agreement with Piaget’s theory of cognitive development which states that development is a methodical and logical process that occurs in distinct stages (Feldman 2004; James and Nelson 1981). The overall process is influenced by the quality of experiences in the physical and social world, together with the drive for equilibrium. Equilibrium is the balance between the process of assimilation and accommodation, where assimilation is the fitting of new information into an existing mental structure and accommodation is the creation of new schemata (knowledge structures) or modification of an existing schema (Thompson 1999). Research is deficient regarding the applicability of the Housen model in science education. However the model has sensible theoretical basis.

The CVM can also be understood according to the dual coding and the constructivist theories. According to the dual coding theory (Wastelinck et al. 2005; Clark and Paivio 1991), the human cognitive structure has two mental processing systems, a verbal and non-verbal system (also known as auditory-verbal and visual-pictorial channel respectively). The theory states that human cognition is capable of dealing with verbal or linguistic and non-verbal knowledge as independent knowledge structures in their own right (Wastelinck et al. 2005; Clark and Paivio 1991). Through referential connections, the two systems work together to construct and integrate mental visual models which are then memorized and stored in the long term memory (Clark and Paivio 1991).

However, according to the limited capacity theory the working memory has a limited capacity for holding and manipulating information (Mayer and Anderson 1992). This limited capacity suggests that if the visual-pictorial channel is presented with enormous visual information it can be overwhelmed and fail to integrate information properly (Whelan 2007; Mayer and Anderson 1992). The resulting overload leads to an inability to process new information effectively and hence, the construction of mental visual models is compromised. Therefore, effective CVM depends very much on the amount of information presented to each of the cognitive channels. McClean et al. (2005) present evidence showing that student retention of content material in biology education is improved when such content is presented via a lecture coupled with the animations and when an animation is used as an individual study activity. Their argument is that animations and the lecture allow students to process information using the two channels. This is further enhanced when students work with the animation at their preferred pace which ensures that their cognitive structures are not overwhelmed.

**Figure 5:**
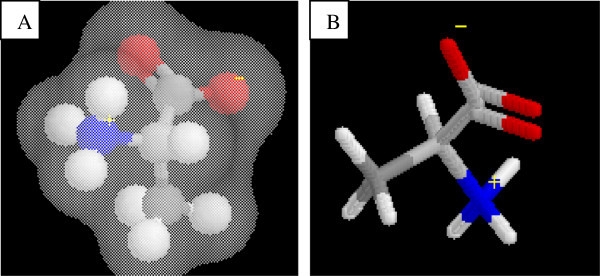
**Two ERs where students were required to observe the differences in orientation of the amino acids (adapted from Mnguni 2007). A)** A depiction of Alanine as a ball-and-stick model with an electron cloud, **B)** a depiction of alanine using a stick model

Mnguni (2007) argues that the ability to recognize (which is IVM) and interpret visual models of a concept that is represented in different orientations is an example of CVM. In his study he found that students had difficulties recognising amino acids, through analysing visual models and integrating prior knowledge, when these models were placed at different orientations and presented using different symbols (Figure 5). What this example illustrates also is that visualization cannot be understood in isolation, instead there is always a certain degree of content linked to it.

### Externalization of visual models

Visualization also involves EVM which is expressing mental visual models (which occurs in the mind) as external visual models in the form of drawings for instance or verbal descriptions. According to Rundgren et al. (2010) external visual models produced by students can be classified into three levels. These are the macroscopic level, the microscopic level, and symbolic level. In the macroscopic level students attempt to produce a visual model of the phenomenon as they directly experienced it through any of their senses. In the microscopic level, students attempt to produce a visual model of phenomena as they exist in nature, even though such phenomena cannot be observed directly with the naked eye, meaning that students may not have any previous direct experience with the phenomena. In the symbolic level the visual model produced by students is a qualitative abstraction such as a mathematical model which is used to represent phenomena. Examples include a chemical equation of glucose metabolism which attempts to explain a metabolic process which cannot be directly observed.

Stokes (2002) suggests that the production of a visual model (i.e. EVM) is a process where a student expresses a mental visual model into an external visual model. West (1997) argues that some students may attempt to express the mental model externally in the exact way that it occurs in their mind, even though this may be difficult to do. In other cases however, a mental visual model may be expressed externally in a different format to that in which it occurs in the mind, e.g. textually, numerically or verbally.

It is critical to state that expressing mental models as external visual models depends on various factors such as intelligences. For instance, when drawing a diagram, the bodily-kinesthetic intelligence would play a role as it determines the way one moves his/her hand and fingers. Also, logical-mathematical intelligence would play a role in the expression of mental visual models numerically. In addition, the spatial/visual intelligence as well as linguistic intelligence are critical for the expression of visual mental models in the verbal form. As a result, it may be suggested that the manner in which students express visual models depends highly on their cognitive and physical abilities.

To test for EVM, Mnguni (2007) asked students to study (i.e. IVM and CVM) part of a biochemical process (with parts intentionally removed from the visual model) and then predict and express the missing parts of such a process in a drawing. A visual model of protein synthesis from mRNA using codons was used in this regard. Results showed that some students (58%) were not able to use available information to make inferences about the subsequent steps of a biochemical process (i.e. all part of CVM).

**Figure 6:**
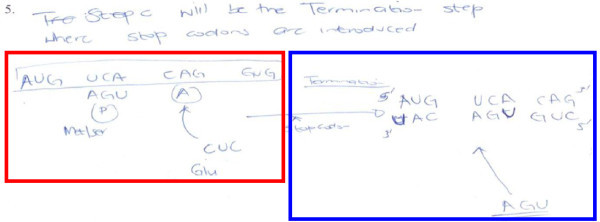
**An example of a student generated diagram where the student has difficulties inferring or predicting (adapted from Mnguni** 2007 **).**

In Figure 6, student 2P32 was able to recognise the process and use correct terminology in describing it (i.e. the use of terms “termination” and “stop codon” in the response). As shown in the red box (Figure 6), the student was also able to re-present the information already depicted in the visual model. However, looking at the blue box, the student fails to use the extracted information (red box) to express the subsequent stages (which were missing in the blue box) and hence, provides an incorrect outcome of the process. This example demonstrate a case where students need to use content knowledge (from prior knowledge) to produce visual models by way of externalizing visual models.

### Application of the IVM, CVM and EVM model in science education

The above discussion presents a detailed theoretical account of the cognitive process of visualization which demonstrates the complexity of visualization. It is evident that visualization is not a linear process. Instead various interconnected actions occur, which are driven by the need to generate knowledge. It emerges that these are dependent of prior knowledge which is used to construct new knowledge. Given this, it is critical therefore that visual literacy education be formalized in a similar way to other competencies such as content literacy (McKenna and Robinson 1990), academic communication literacy (Spektor-Levy et al. 2008) and science literacy (Van Eijck and Roth 2010). Furthermore, the author argues that teachers need to be alerted to the complexity of visualization and its significant, in order to assist them develop tools with which learning difficulties could be minimized. Science education teachers need to be able to identify the source of a learning difficulty associated with visualization and use appropriate remedial strategies to assist students. For example, it is important that teachers understand the significance of each of the stages of the theoretical cognitive process of visualization so that they can design relevant instructional strategies to facilitate learning. Given this, it is important that the visualization skills be identified within the context of visual literacy for science education. To this end, the following applications need to be considered in order to better understand, teach or study visual literacy in science education:

- IVM is characterized by three main components, namely, low, middle and high level in relation to the cognitive effort applied to comprehend visual information. The significance of these in terms of further understanding, teaching or studying visual literacy in science education is that:
  i) Visualization tasks relevant to each level must be identified explicitly so that teaching and learning could encompass a gradual move from low level to high level EMV. These tasks must be integrated to relevant content knowledge and learning objectives.
  ii) Visual models need to be classified according to their IVM levels in relation to tasks and context.
  iii) Students’ ability to operate with visual models from each level needs to be investigated and documented. This will ensure that students are not overwhelmed or underwhelmed with visual materials outside their zone of proximal development.
- CMV relates mainly to the integration of prior knowledge with new knowledge through various cognitive processes such as chunking and organizing. Consequently, in order to better understand, teach or study visual literacy in science education, the following need to be done:
  i) Students’ already existing cognitive skills and prior knowledge (including misconceptions) related to visualization must be explored, in order to facilitate learning through visual models.
  ii) Students’ preferred visual learning styles must be considered during curriculum and instructional design and development.
  iii) Activities that could help students to better construct knowledge using visual models need to be integrated into curricula.
- EVM is mainly about students’ ability to communicate knowledge from their working memory by way of externalizing cognitive visual models. This could be facilitated by:
  i) Determining students’ ability to externalize visual models, in order to ensure that tasks do not require students to produce visual models that are beyond their abilities.
  ii) Incorporating into curricula visualization tasks that could improve students’ ability to communicate visually at the different levels, i.e. macro, micro and symbolic levels.

The author also proposes further research into the applicability of the theoretical cognitive process of visualization discussed in this article. Empirical research is also necessary to determine the significance of visual literacy in science education.

## References

[CR1] Allen RE (1990). The concise Oxford dictionary of current English.

[CR2] Arsad NM, Osman K, Soh TMT (2011). Instrument development for 21st century skills in Biology. Procedia Soc Behav Sci.

[CR3] Behrens R (1984). Design in the visual arts.

[CR4] Bottomley S, Chandler D, Morgan E, Helmerhorst E (2006). jAMVLE, a new integrated molecular visualization learning environment. Biochem Mol Biol Educ.

[CR5] Brill JM, Kim D, Branch RM: *Visual literacy defined: the results of a Delphi study: can IVLA (operationally) define visual literacy?*. 2000. [*Paper presented at the International Visual Literacy Association, Ames, IA*]

[CR6] Burton L (2004). Helping students become media literate. Workshop’s paper. Australian School Library Association (NSW) Inc. 5th State Conference.

[CR7] Clark JM, Paivio A (1991). Dual coding theory and education. Educ Psychol Rev.

[CR8] DeSantis K, Housen A (2000). A Brief Guide to Developmental Theory and Aesthetic Development.

[CR9] Dori YJ, Barak M (2001). Virtual and physics molecular modelling: fostering model IVM and spatial understanding. Educational Technology and Society.

[CR10] Feldman DH (2004). Piaget’s stages: the unfinished symphony of cognitive development. New Ideas Psychol.

[CR11] Healey CG (2005). Perception in visualization.

[CR12] Housen A (1992). Validating a Measure of Aesthetic Development for Museums and Schools. ILVS (International Laboratory for Visitor Studies). Review.

[CR13] James HL, Nelson SL (1981). A classroom learning cycle: using diagrams to classify matter. J Chem Educ.

[CR14] Kawahara J-I, Yokosawa K (2001). Pre-attentive perception of multiple illusory line-motion: a formal model of parallel independent-detection in visual search. J Gen Psychol.

[CR15] Koedinger KR, Anderson JR (1990). Abstract planning and perceptual chunks: elements of expertise in geometry. Cogn Sci.

[CR16] Mast FW, Ganis G, Christie S, Kosslyn SM (2003). Four types of visual mental imagery processing in upright and tilted observers. Cogn Brain Res.

[CR17] Mayer RE (2002). Rote versus meaningful learning. Theory Into Practice.

[CR18] Mayer RE (2003). Learning and instruction.

[CR19] Mayer RE, Anderson RB (1992). The instructive animation: helping students build connections between words and pictures in multimedia learning. Journal of Educational Psychology.

[CR20] McClean P, Johnson C, Rogers R, Daniels L, Reber J, Slator BM, Terpstra J, White A (2005). Molecular and cellular biology animations: development and impact on student learning. Cell Biol Educ.

[CR21] McKenna MC, Robinson RD (1990). Content literacy: a definition and implications. J Read.

[CR22] Mnguni L (2007). Development of a taxonomy for visual literacy in the molecular life sciences.

[CR23] Mullet K, Sano D (1995). Designing visual interfaces: communication oriented techniques.

[CR24] Novick LR, Catley KM (2007). Understanding phylogenies in biology: the influence of a gestalt perceptual principle. J Exp Psychol Appl.

[CR25] Rundgren C, Chang Rundgren S, Schönborn KJ (2010). Students’ conceptions of water transport. J Biol Educ.

[CR26] Schönborn KJ, Anderson TR (2009). A model of factors determining students’ ability to interpret external representations in biochemistry. Int J Sci Educ.

[CR27] Schönborn KJ, Anderson TR (2010). Bridging the educational research-teaching practice gap – foundations for assessing and developing biochemistry students’ visual literacy. Biochem Mol Biol Educ.

[CR28] Spektor-Levy O, Eylon B, Scherz Z (2008). Teaching communication skills in science. Teach Teach Educ.

[CR29] Stevenson SB, Roorda A (2005). Miniature eye movements measured simultaneously with ophthalmic imaging and a dual –Purkinje image eye tracker. J Vis.

[CR30] Stokes S (2002). Visual literacy in teaching and learning: a literature perspective. Electronic Journal for the integration of Technology in Education.

[CR31] Thompson PW, Steffe LP, Gale J (1995). Constructivism, cybernetics and information processing: implications for technologies of research on learning. Constructivism in education.

[CR32] Thompson JM (1999). Enhancing Cognitive Development in College Classrooms: A Review. Journal of Instructional Psychology.

[CR33] Turner JL, Dankoski ME (2008). Objective Structured Clinical Exams: A Critical Review. Fam Med.

[CR34] Van Eijck M, Roth W (2010). Theorizing scientific literacy in the wild. Educ Res Rev.

[CR35] Van Schoren J (2005). Levels of perception.

[CR36] von Glasersfeld E, Steffe LP, Gale J (1995). A constructivist approach in teaching. Constructivism in education.

[CR37] Wastelinck KD, Valcke M, Craene BD, Kirschner P (2005). Multimedia learning in social sciences: limitations of external graphical representations. Computers in Human Behaviour.

[CR38] West TG (1997). In the mind’s eye.

[CR39] Whelan RR (2007). Neuroimaging of cognitive load in instructional multimedia. Educ Res Rev.

